# Artificial Intelligence Tools in Myocardial Infarction Prognosis: Evaluating the Performance of Machine Learning and Deep Learning Models

**DOI:** 10.2174/011573403X380162250706094642

**Published:** 2025-07-11

**Authors:** Cyntia Szymańska, Artur Baszko

**Affiliations:** 1 2^nd^ Department of Cardiology, Poznan University of Medical Sciences, Poznań, Poland

**Keywords:** Risk stratification, AI tools, risk factors, prognostic tools, endpoints, machine learning (ML)

## Abstract

In clinical practice, mortality risk assessment in patients with myocardial infarction often relies on scales such as GRACE and TIMI. However, these scales were developed based on cohorts assembled many years ago. Since then, numerous changes have occurred, ranging from shifts in MI patient profiles to the introduction of new antiplatelet medications and the adoption of more restrictive lipid therapy targets. To address this issue, researchers are working to develop new stratification tools. Artificial intelligence (AI), which finds applications in nearly every area of medicine, also presents solutions to this problem. This review includes sixteen papers that contain machine learning and deep learning models used to prognosticate mortality risk at different points. Machine learning (ML) models, such as random forest, gradient boosting, and support vector machines, have demonstrated good to excellent performance. However, no single algorithm appears to be top-performing. Although artificial neural networks are considered one of the most promising algorithms, they do not invariably outperform other ML methods. The adaptability of AI models to various scenarios and their ability to handle complex datasets reassures us of their potential in cardiology. Concerning variables that influence the risk of mortality, most are well-established factors, such as age, left-ventricular ejection fraction, lipid parameters, and B-type natriuretic peptide. Additionally, less apparent indicators include platelet parameters, neutrophil count, and blood urea nitrogen. In conclusion, utilizing AI-based models in myocardial infarction risk stratification presents a significant opportunity to develop effective and tailored tools.

## 1 INTRODUCTION

Myocardial infarction (MI) consistently accounts for an important part of deaths and is considered an essential public health issue. Its global prevalence is estimated to be 3.8% in the age group below 65 years and 9.5% over 65 years [1]. Despite progress in the treatment of MI, mortality remains a significant concern. In 2019, 30-day mortality rates following hospital admission varied considerably across countries, ranging from 3.2 per 100 admissions for Denmark to 5.5 for Iceland and up to 17.0 for Latvia [2]. Researchers have sought tools to assess the mortality risk in MI cases.

In clinical practice, the GRACE risk score is a tool used to assess the risk of death or non-fatal myocardial infarction in a hospital and the subsequent six months [3]. The calculations were done using the Cox regression model and assessed by the area under the curve (AUC) value. The final score considers nine factors: age, congestive heart failure, peripheral vascular disease, systolic blood pressure (SBP), Killip class, initial serum creatinine concentration, positive initial cardiac markers, cardiac arrest on admission, and number of leads with ST deviation. This tool has been widely validated on external data in many studies [4, 5].

A few years later, this scale was updated [6]. The new scale version assumes non-linear dependencies between variables and risk. Additionally, instead of computing numerical values, the latest version provides an electronic version with absolute percentage risks. Non-linear associations were found for SBP, pulse, age, and creatinine level. Substitutes for creatinine and Kilip class can be utilized to make the scale easier when these factors are unavailable. Its performance is slightly better concerning in-hospital and 6-month mortality.

Another valuable tool for assessing the risk of myocardial infarction (MI) is the Thrombolysis in Myocardial Infarction (TIMI) Score [7]. This scoring system was developed using data from the unfractionated heparin (UFH) arm of the TIMI 11B trial with multivariate logistic regression analysis. The identified significant risk factors include various variables, such as age (over 65 years), presence of three or more risk factors for coronary artery disease (positive family history, hypertension, hypercholesterolemia, diabetes, or current smoking), known coronary artery disease with significant stenosis (≥50%), severe anginal symptoms (defined as experiencing two or more anginal events in the preceding 24 hours), recent aspirin use within the last seven days, ST-segment deviation (≥0.05 mV), and elevated serum cardiac markers indicating necrosis. Each factor contributes to the risk score, assigning one point for each factor that is present.

Another worth mentioning score is a model based and validated on The Acute Coronary Treatment and Intervention Outcomes Network (ACTION) Registry^®^-Get With The Guidelines (GWTG)™ [8]. It was a cohort that included patients who had either STEMI or NSTEMI and were admitted to one of more than 300 hospitals in the United States and included factors obtained age, baseline serum creatinine, systolic blood pressure, baseline troponin ratio, heart rate at admission, heart failure/shock on presentation, electrocardiography (ECG) findings, and prior peripheral artery disease. Points are assigned based on specific values for each factor, and the probability of death can be determined from a chart using the final score.

However, these scales were developed based on data from many years ago - GRACE includes patients enrolled between April 1999 and September 2005, TIMI between August 1996 and March 1998, and ACTION between January 2007 and September 2008. Since that time, many aspects of MI have changed. First of all, the patient profile differs substantially. Mercado-Lubo *et al.* (2019) analyzed changes in the characteristics of patients with MI between 2001 and 2011 [9]. Patients in the latter years of the study were younger, had lower serum troponin levels, and experienced shorter hospital stays than patients from the earliest study years.

Another crucial aspect to consider is the development of novel antiplatelet drugs after the publication of the papers mentioned above. Prasugrel gained approval in the European Union and the United States of America in 2009 [10], while ticagrelor gained approval in 2010 and 2011 [9], respectively. Also, the goal of lipid therapy has been systematically reduced from a level of low-density cholesterol lower than 100 mg/dL [10], later 70 mg/dL [11], up to lower than 55 mg/dL in current guidelines [12]. All these aspects may gradually reduce the usefulness of these scales.

## 2 MACHINE LEARNING

In recent years, the significance of artificial intelligence in medicine and its branches - machine learning and deep learning - has been steadily increasing [13]. These technologies enable the development of novel algorithms capable of transforming how we assess risk.

Machine learning (ML) is a branch of artificial intelligence that creates algorithms that work similarly to how people learn. Thanks to exposure to data, its accuracy is constantly improving. Using algorithms may result in finding more complex dependencies that cannot be elucidated with simple mathematical equations. It might help discover previously overlooked but important factors.

There are three main approaches in machine learning: supervised, unsupervised, and reinforcement machine learning [14]. In unsupervised learning, algorithms search for patterns in unlabeled data and seek hidden structures without human intervention [15]. This type of learning is precious in medical data analysis because it bypasses the need for costly and time-consuming data labeling, a significant bottleneck in clinical applications. While unsupervised learning may not always match the accuracy of supervised methods, it enables exploratory data analysis, clustering, and dimensionality reduction, which can be used to discover subgroups and insights that inform research and patient care [16].

In supervised machine learning, input data are labeled. The dataset is divided into at least two groups: training and testing sets [17]. Using the training set, the algorithm adjusts its parameters [18]. Then, we can evaluate the learning process with the testing set. Additionally, a validation dataset may be separated, which is used to tune hyperparameters and is unique for each type of algorithm [19]. These algorithms solve both classification and regression problems [17]. It might apply to the distinction between healthy and sick individuals in medicine.

Reinforcement learning, the third type of machine learning, operates through the interaction of a model with its environment [20]. The agent learns iteratively, refining its actions through trial and error to maximize cumulative rewards. Key components integral to reinforcement learning include the agent (learner or decision-maker), environment (the external system with which the agent interacts), state (the current situation or configuration of the environment), action (the decisions or choices made by the agent), reward (scalar feedback signals provided by the environment to the agent), and policy (determination of action in current state) [21]. Interactions between these components allow the model to solve complex problems.

### 2.1 Topological Data Analysis

Topological Data Analysis (TDA) is generally considered unsupervised machine learning. TDA is a set of techniques rooted in topology that is used to understand the shape and structure of complex data [22]. It strongly emphasizes comprehending the “shape” of data and identifying patterns, such as loops and clusters, that hold true at many scales. The primary tool in TDA, persistent homology, tracks these features as the scale changes, identifying structures that are robust *versus* those that may be noise [23, 24]. TDA is beneficial when examining data with complex structures, where conventional techniques could miss underlying links or patterns.

### 2.2 Logistic Regression and Regularization

It is a method at the intersection of statistics and machine learning. It estimates a binary value based on single or multiple variables [25]. The result of this method is the logarithm of the odds of the event occurring. In medical research, we can apply it to mortality analysis [26-28].

When the number of variables is vast, we can turn to variants with regularization [16]. This method minimizes the risk of overfitting the model, which occurs when the model tries to fit every value [29]. For this reason, the model performs very well on the training data but poorly on test or real-world data [30].

There are several types of regularization, with two common approaches being ridge (L2) and lasso (L1) regularization [31]. Ridge regularization incorporates the square of the model’s weights into the loss function, penalizing large weights and encouraging smaller, non-zero values [32]. Lasso regularization incorporates the absolute value of the model’s weights, promoting sparsity by reducing some coefficients to exactly zero [33]. Elastic net combines both types of regularization, which can provide more balanced results [34].

### 2.3 Tree-based Models

A decision tree is a model structured like a tree. It comprises a root node, which initiates the decision-making process, followed by internal nodes representing decisions based on features [35]. Each branch emanating from these nodes leads to subsequent nodes or leaves, which signify the outcome or class label of the decision process [36].

Random Forests (RF) involve assembling numerous decision trees generated during the model training and used for predicting classes or values [37]. The algorithm earns its “random” designation through data sampling and feature subsets' random selection. The outcome is a composite of predictions made in each tree [38].

Bootstrap aggregating, or bagging, combines multiple individual models' predictions [39]. This algorithm creates various subsets of data using random sampling with replacement from the original training set [40]. It should be the same size as the original data set, so some data might appear more than once in a subset, while others may not. Then, a separate model is trained on every subset independently of the other. It is used to make predictions, which are combined in the next step. In classification tasks, majority voting is used, where the class predicted by the most models is selected as the final output [41]. In regression tasks, averaging is typically used, where the predictions of all models are averaged to produce the final result [42]. This process helps to reduce overfitting and improve the generalization of the model.

Gradient Boosting (GBM) is a potent machine-learning approach that sequentially integrates multiple weak learners (models that are slightly better than random guesses) to construct a resilient predictive model [43]. To prevent overfitting, the learning rate parameter is employed [44]. With values ranging from 0 to 1, it regulates how much each weak learner affects the final prediction. Its notable advantage lies in its interpretability, unlike black-box models, making it easier to discern the factors influencing the final model. Practical implementations of this technique include AdaBoost [45], eXtreme Gradient Boosting (XGBoost), and CatBoost.

XGBoost stands as a prominent algorithm embodying the principles of gradient boosting. Its distinguishing features from other methods encompass regularization options, automated feature selection capabilities, and an additional randomization parameter [46]. CatBoost is another boosting implementation. Its main advantages are handling categorical variables without pre-processing and automatically managing missing data. Also, it prevents overfitting due to numerous mechanisms, such as regularization, learning rate annealing, and early stopping [47].

### 2.4 Support Vector Machines

Support Vector Machines (SVMs) are algorithms that solve complex classification and regression challenges. They are particularly effective when the number of variables exceeds the number of cases. At its core, SVM identifies a hyperplane, a decision boundary that effectively separates the two classes [48]. This hyperplane aims to maximize the margin distance between classes while minimizing classification errors. By doing so, SVM achieves robust performance in various domains, ensuring accurate and reliable predictions even in high-dimensional spaces where traditional methods may struggle [49].

SVM types are categorized as linear and non-linear. The first uses a linear hyperplane as the decision boundary, suitable for datasets with linear separability [50]. Non-linear SVMs handle non-linearity in data separation by utilizing kernel functions. These kernel functions, such as polynomial [51], Gaussian [52], and sigmoid [53], enable SVMs to map input features into higher-dimensional spaces, facilitating the efficient separation of classes in complex, non-linear datasets [54].

### 2.5 Naive Bayes

Naive Bayes classification is grounded in Bayes' theorem, incorporating a “naive” presumption of feature independence [55]. It predicts the probability of an instance belonging to each class and selects the one with the highest probability, disregarding inter-feature relationships. During training, it learns the probability distribution of features for each class from labeled data, then computes probabilities and makes predictions accordingly [56]. Naive Bayes is a family of classifiers, not a single algorithm, with variants tailored to different data types. The examples are Gaussian Naive Bayes (for continuous features) [57], Multinomial Naive Bayes (for discrete features) [58], and Bernoulli Naive Bayes (for binary features) [59].

### 2.6 Artificial Neuron Network and Deep Learning

An artificial neural network (ANN) is a computational model inspired by the architecture and functioning of the human brain [60]. Composed of interconnected nodes organized into layers, ANNs resemble biological neural structures. These layers include an input layer that receives initial data, one or more hidden layers for intermediate processing, and an output layer that produces the final result [61]. Connections between nodes carry weights that control the strength of information transfer and directly impact neuron outputs. The flow of information typically proceeds in one direction, from the input to the output layer, with each node applying an activation function-often non-linear-to the weighted sum of its inputs, enabling the modeling of complex, non-linear relationships in data [62, 63].

ANNs learn by iteratively adjusting connection weights through a training process that involves exposure to labeled data and updates based on discrepancies between predicted and actual outputs [64]. This process underpins deep learning (DL), which leverages the multi-layered architecture of ANNs to tackle complex problems, including those in medicine. Notable types of ANNs include convolutional neural networks (CNNs) and recurrent neural networks (RNNs).

CNNs are a specialized type of feed-forward neural network that incorporates regularization techniques. They automatically learn to extract features through the optimization of convolutional kernels. Designed specifically for processing and analyzing data with a grid-like structure, such as images, CNNs excel at tasks like image classification and object detection. They consist of three main layers: the Convolutional Layer, the Pooling Layer, and the Fully Connected (FC) Layer [65].

RNNs process data across multiple time steps, making them well-adapted for modeling and processing text, speech, and time series [66]. These models are employed for various tasks, such as classification, regression, and segmentation, across multiple domains, including diagnostic imaging and genomic studies.

While ANNs offer significant capabilities, their complexity often leads to their characterization as 'black-box' models, where the inner workings are not easily interpretable. This has raised concerns, especially in medical applications where understanding the rationale behind decisions is critical for clinical validation. Although interpretability has historically been a limitation, recent methods such as SHAP (Shapley Additive Explanations) [67], LIME (Local Interpretable Model-agnostic Explanations) [68], and LRP (Layer-wise Relevance Propagation) [69] now provide partial insights into the decision-making processes of DL models. Including these interpretability tools could improve transparency in medical decision-making and potentially enhance the accuracy (*e.g*., AUC values) of DL models by fostering clinician trust and supporting diagnostic processes. Factors like computational resource requirements and ongoing model maintenance remain relevant limitations that should be considered alongside interpretability.

## 3 MACHINE LEARNING MODELS' PERFORMANCE IN MYOCARDIAL INFARCTION MORTALITY

### 3.1 Supervised Machine Learning Models

Artificial intelligence is increasingly discovering new applications with each passing year. Researchers have also tried implementing these algorithms to predict risks or mortality rates. In recent years, several studies have emerged aiming to compare these AI-driven approaches with traditional clinical tools and to identify additional crucial factors that can help improve them. Most often, researchers use supervised machine learning methods.

We included 16 original papers focusing on mortality prediction in MI using various machine-learning methods. The original papers were identified through a detailed search strategy conducted in PubMed in April 2024, using the keywords “machine learning AND myocardial infarction mortality”; inclusion criteria required using at least one of the machine learning algorithms to build the model. The size of the participant groups in these studies varied considerably, ranging from 438 to 175,895 individuals. The furthest-in-time cohort started in 2005 (Sherazi *et al.*, 2020 [70]). The number of variables in models ranges from 13 to 84.

Furthermore, 3 of them compared the results of algorithms to previously used scores, and three studies applied the dimensionality reduction method of Recursive Feature Elimination (RFE). At the same time, one addressed class imbalance through methods like the Synthetic Minority Oversampling Technique (SMOTE). Also, deep learning algorithms were included in 6 studies. All included models are listed in Table **1**, detailing the number of cases, assessed mortality points, and the area under the curve (AUC) of the top-performing model.

The value of AUC in all included studies is good or excellent. Four studies compared ML models with traditional clinical scores, such as the GRACE and TIMI scores, often demonstrating superior performance. Zhu *et al.* (2024) [71] achieved the highest AUC using a bagging algorithm among the seven ML models tested. Similarly, Lee *et al.* (2020) employed a combined approach featuring two bootstrap decision forest models and one boosted tree model, optimized using principal component analysis (PCA) and recursive feature elimination (RFE), which achieved robust performance [72].

Interestingly, while some studies indicate that complex models, such as deep neural networks (DNN), do not always outperform non-DNN methods, others, including Sherazi *et al.* (2020) [70] and Khera *et al.* (2021) [73], have found that tree-based models, like GBM and XGBoost, perform comparably or even better than DNN. This highlights the importance of selecting appropriate algorithms and pre-processing methods, as demonstrated by Oliveira *et al.* (2023) [74], who experimented with various feature sets and pre-processing techniques (*e.g.*, SMOTE for addressing class imbalance).

A recurring issue in the reviewed literature is the inconsistent reporting of significant variables. Despite the availability of interpretability techniques like SHAP, only half of the studies identified crucial model features. One of the most consistently highlighted variables across mortality prediction models is age. Its significance is observed across various studies, including those by Aziz *et al.* (2021) [75], who identified age as a crucial predictor in in-hospital, 30-day, and 1-year mortality models. Similarly, Lee *et al.* (2020) [72] found age to be among the top-ranked variables when applying Recursive Feature Elimination (RFE), and it was also a key factor in the models analyzed by Mansoor *et al.* (2017) [76] and Sherazi *et al.* (2020) [70].

Cardiac function variables, such as Killip class, left ventricular ejection fraction (LVEF), and heart rate, consistently emerge as significant predictors of mortality. Aziz *et al.* (2021) [75] identified Killip class as one of the most critical variables for in-hospital mortality, while Lee *et al.* (2020) [72] identified LVEF and initial Killip class as two of the top 17 variables identified through recursive feature elimination (RFE) and in studies focused on machine learning models, such as those by Sherazi *et al.* (2020) [70], cardiac-related metrics, including creatinine levels and coronary angiography, also appeared prominently. This alignment suggests that both functional cardiac markers and indicators of cardiac stress are vital for predicting mortality risk. Indicators of acute events, such as out-of-hospital cardiac arrest (OHCA) and cardiogenic shock, are essential for predicting short-term mortality. Chen *et al.* (2023) [76] and Zhu *et al.* (2024) [71] both identify OHCA and cardiogenic shock as key features in their models, underscoring the significant immediate risk these conditions pose. Mansoor *et al.* (2017) [77] also highlighted the importance of cardiogenic shock in multivariable analyses, suggesting that acute cardiac events have a significant impact on mortality outcomes.

B-type natriuretic peptide (BNP) and related markers, such as NT-proBNP, consistently rank among the most predictive biomarkers for mortality. Song *et al.* (2023) [78] identified BNP as one of the top five variables using SHAP analysis, while Jeong *et al.* (2024) [79] highlighted NT-proBNP as a predictor of both 1-year and 3-year mortality using a Random Forest model. Additionally, Zhu *et al.* (2024) [71] demonstrated the relevance of BNP through BorutaSHAP, reinforcing its role as a crucial indicator of heart failure severity and patient prognosis. Inflammatory and metabolic markers, including C-reactive protein (CRP), triglycerides (TG), uric acid, and blood urea nitrogen (BUN), remain significant. Lee *et al.* (2020) [72] and Song *et al.* (2023) [78] both reported CRP and TG as essential factors. In contrast, Zhu *et al.* (2024) [71] underlined the importance of BUN, particularly in models evaluating the impact of renal function on cardiovascular mortality.

Procedural variables, including percutaneous coronary intervention (PCI) and coronary angiography, along with treatment-related factors like statin use and discharge medication administration, significantly affect mortality predictions. Sherazi *et al.* (2020) [70] reported that PCI during hospitalization and the administration of acetylsalicylic acid at discharge were crucial in the best-performing Gradient Boosting Machine (GBM) model. Jeong *et al.* (2024) [79] also emphasized the importance of PCI and antiplatelet maintenance for predicting one-year mortality.

The relationship between sample size and model performance is nuanced. Larger datasets, such as those used by Khera *et al.* (2021) [73] (993 905 cases) and Kwon *et al.* (2019) [80] (68 528 cases), consistently demonstrate high AUC values (0.886 and 0.905, respectively), suggesting that larger sample sizes can facilitate robust model training. However, smaller datasets, such as Chen *et al.* (2023) [76] (438 cases), and Oliveira *et al.* (2023) [74] (1 749 cases), also achieved high AUC values (0.93 and 0.89), indicating that model architecture and feature selection may play more critical roles than mere data volume.

### 3.2 Unsupervised Machine Learning Approaches

A few studies have also employed unsupervised machine learning methods to analyze MI mortality. Hathaway *et al.* (2024) [81] conducted a study using echocardiography images from the apical 2, 3, and 4-chamber views. The images were taken from a retrospective cohort of patients with STEMI and NSTEMI. Using topological data analysis (TDA), they identified three distinct clusters associated with different risk levels for 1-year mortality (high-risk, intermediate-risk, and low-risk). The risk levels were calculated using the GRACE score.

To our knowledge, there is no approach to reinforcement learning in myocardial infarction prognosis.

## 4 DISCUSSION

ML and DL have emerged as transformative medical tools, offering significant advancements in risk stratification across a diverse range of medical conditions. AI models offer a significant advantage over traditional risk scoring methods by incorporating unobvious variables, such as platelet parameters, allowing for more personalized risk predictions that traditional static scores may overlook. These technologies have shown great promise in addressing complex issues across a wide range of aspects, including obesity [82], renal dysfunction [83], cancer [84, 85], and diabetes mellitus [86]. In cardiology, they are used for predicting cardiovascular risk [87, 88], assessing left heart structure and function [89-91], and evaluating the risk of atrial fibrillation [92]. These methods have the potential to explore large datasets systematically. They can reduce costs and improve the speed of getting data analysis results.

There is no leading algorithm for non-deep learning ML models. The main difficulty in comparing the performance of algorithms is the authors' subjective selection of models and features. Some studies focus solely on comparing different ML models, while others incorporate DL models, and others compare them with currently used scales. Only a few studies (Aziz *et al.*, 2021 [75]; Khera *et al.*, 2021 [73]) discuss why these algorithms were chosen. However, identifying the optimal ML algorithm remains ambiguous, as studies highlight varying top performers. Support vector machines, random forests, or boosting models have been particularly dominant in certain studies. Different algorithms should be employed when selecting the optimal model.

Regarding significant variables from studies where they are available, it reveals that while age, cardiac function metrics (such as BNP and Killip class), and procedural interventions (such as PCI) are universally important, the inclusion and ranking of variables often vary depending on the modeling technique and the specific patient population. The challenge lies not only in identifying the most predictive variables but also in understanding how these predictors interact within different clinical contexts and time frames. By looking beyond individual papers and focusing on broader themes, this value-centered approach provides a more nuanced understanding of mortality prediction. It underscores the need for models that can dynamically integrate demographic, clinical, and procedural data, emphasizing the significance of the work in this field.

DL may seem more effective than other ML algorithms, however it does not consistently achieve the best AUC values. (Khera *et. al* (2021), Sherazi *et al.* (2020), Jeong *et al.* (2024)). The most uncommon observation comes from Jeong (2024), where RF emerged as the best-performing model, while even simpler algorithms like NB and LR outperformed ANN. This result emphasizes the critical role of dataset characteristics and model tuning. It also presents an important issue: increased model complexity does not mean better performance, especially when data volume or quality is suboptimal. The reason for this outcome is that deep learning models often underperform on tabular tasks due to the diversity of feature types and scales, as well as irregular and skewed distributions, which tree-based ensembles manage more robustly [93]. However, neural networks can compete with or even surpass traditional methods; notably, a transformer-based tabular foundation model achieves satisfying accuracy on small to medium datasets [94]. ML algorithms are typically less computationally intensive and provide greater transparency, enabling clinicians to understand how input features influence predictions. However, they may struggle to capture complex patterns in high-dimensional data. In contrast, deep learning (DL) models, especially convolutional neural networks, excel at handling intricate data structures, such as images and electrocardiograms, often achieving superior accuracy. This advantage comes with increased computational demands and reduced interpretability. These factors underscore the importance of carefully selecting modeling approaches tailored to specific clinical applications, striking a balance between computational efficiency and the need for model transparency.

These findings point to the importance of aligning model selection with the specific demands of the problem. ML models, such as RF and XGBoost, offer advantages in competitive performance, computational efficiency, and interpretability. In clinical contexts, the transparency of ML models may foster greater trust among healthcare professionals, facilitating their integration into decision-making processes. Its main limitation in medicine is the feasibility of implementing it in clinical practice. Due to their mathematical complexity, they need high computational power to perform. This issue makes it troublesome to convert them to easily manageable clinical practice forms, such as mobile calculators, which physicians can use in everyday practice in hospitals and outpatient clinics.

Moreover, the strength of traditionally used scores is the wealth of papers that prove their efficacy. To ensure that their remarkable performance is not only the result of overfitting but rather a true indication of their efficacy, algorithms with potential clinical utility must undergo extensive and continuous testing. In addition, some variables selected by models are unobvious and necessitate clinical validation, whether they are previously overlooked predictors or just a work of chance. However, the other aspect is the ongoing updating of conventional risk scales, created based on data collected many years ago.

Despite their potential, AI models in cardiology face challenges such as dataset heterogeneity, which can affect model generalizability, variable selection biases, which can endanger robustness, and a lack of consistent external validation, limiting their application across different patient populations.

One of the current evidence gaps is the effect of group size on ML model performance. Unlike classic statistics, there is no clear consensus on the adequate size of the group in ML [95]. Results on the effect of the group size are unclear and show inconsistency [96, 97]. Small training sample sizes may exaggerate ML accuracy due to overfitting or random effects, but large-scale investigations demand more financial resources and time [98].

The main limitations of reviewed studies include sample size, overfitting, and generalizability. For instance, Zhang *et al.* (2024) used a small cohort of 570 patients, increasing the risk of overfitting and limiting applicability to less severe MI cases. Moreover, Khera *et al.* (2021), despite using a large dataset, lacked external validation, raising concerns about the model's generalizability beyond the original cohort. These examples emphasize the importance of using larger, more diverse datasets and incorporating robust external validation to ensure that AI models in cardiology are both accurate and widely applicable.

Future research in machine learning should aim to bridge the gap between technology and clinical practice [99]. Development models should be as simple as traditional scales and trained on up-to-date datasets. They should also be easily and cost-effectively [100] adaptable to transfer data from hospital information systems. Machine learning should concentrate either on personalized risk or population-scaled predictions. Psychosocial factors, not only biometric variables, should receive greater attention.

## CONCLUSION

The use of artificial intelligence-based models in risk stratification for myocardial infarction remains a promising approach. These methods have the potential to provide more accurate and personalized prognostic tools, which have the promise of significantly impacting patient care. While various ML and DL algorithms have demonstrated strong predictive performance, no single method has outperformed others. Further research is needed to validate these models in clinical practice and to integrate them effectively into existing workflows.

## Figures and Tables

**Table 1:** A summary of model performance is included in all review papers.

**References**	**Number of Cases**	**Point of Mortality**	**Number of Variables**	**Included Algorithms**	**Included Scores**	**Dimensionality Reduction**	**Imbalance of Data Reduction**	**AUC of Best** **Performing Model (95% CI)**
**STEMI and NSTEMI included**								
Mohammad *et al.* (2022) [101]	111 558 (training) 27 730 (testing) 30 971 (validation)	1-year	21 (simplified model) 84 (extended model)	ANN	None	None	None	0.85 (0.84–0.85)
Khera *et al.* (2021) [73]	993 905 complete cases	In-hospital	8 continuous variables and 48 categorical variables	LR, XGB, ANN, Meta	None	None	None	XGB and Meta 0.886 (0.882-0.890)
Lee *et al.* (2020) [72]	21 962	1-year	95 variables	ML model combined from: bootstrap decision forest models and 1 boosted tree model	None	PCA, RFE	None	0.918 (0.902-0.935)
Oliveira *et al.* (2023) [74]	1 749	In-hospital	Experiment 1: 25 laboratory findings + 4 variables Experiment 2: 25 from Experiment 1 + 8 additional variables Experiment 3: Experiment 2 + 5 additional variables	LR, DT, RF, GBM, SVM, kNN, GNB, AdaBoost, SGD	None	RFE	SMOTE	Experiment 1: GNB with feature selection + SMOTE 0.79 (not available) Experiment 2: SVM with feature selection + SMOTE 0.81 (not available) Experiment 3: kNN + SMOTE 0.89 (not available)
Song *et al.* (2023) [78]	5 240	1-year	44	SVM, XGB, AdaBoost, kNN, RF, CatBoost, DT, LDA	None	None	None	LDA 0.83 (not available)
Zhu *et al.* (2024) [71]	5 836	In-hospital	67	LR, RF, XGBt, SVM, MLP, GBM, Bagging	None	None	None	Bagging 0.932 (0.918- 0.946)
Sherazi *et al.* (2020) [70]	8 227	1-year	2 continuous variables 43 categorical, 4 discrete	RF, LR, GBM, ANN	GRACE	None	None	GBM and ANN 0.898 (not available)
Jeong *et al.* (2024) [79]	9 661	1-year, 3-year	77	ANN, DT, LR, NB, RF, SVM	None	None	None	RF 0.8738 (not available), 0.7857 (not available)
**STEMI and NSTEMI were included in two separate groups**								
Kwon *et al.* (2019) [80]	22 875	In-hospital	13	ANN, RF, LR	GRACE, ACTION, TIMI	None	None	ANN STEMI 0.905 (0.902-0.909) NSTEMI 0.870 (0.865-0.876)
Kasim *et al.* (2022) [102]	68 528 all cases 13 190 complete cases	In-hospital	54	RF, SVM, LR, ANN	None	None	None	DL (SVM selectedvar) STEMI 0.96 (0.95,0.96) NSTEMI 0.95 (0.94,0.95)
**STEMI only included**								
Aziz *et al.* (2021) [75]	6 299 (in-hospital) 3 130 (31-days) 2 939 (1-year)	In-hospital, 31-days, 1-year	50	RF, SVM, LR	TIMI	SBE, RFE	None	SVM varImp-SBE-SVM 0.88 (0.846–0.910), 0.90 (0.870–0.935), 0.84 (0.798–0.872)
Chen *et al.* (2023) [76]	438 (patients with diabetes)	In-hospital	43	RF, CatBoost, GNB, LR, GBC, XGB	None	None	None	CatBoost 0.93 (0.925–0.932)
Mansoor *et al.* (2017) [77]	9 637 (women)	In-hospital	11 (LR), 32 (RF), 32 (combined), 17 (reduced)	LR, RF	None	None	None	LR 0.84 (not available)
Hadanny *et al.* (2021) [103]	2 782	30-days	32 (full model), 10 (simple RF model)	RF	GRACE	None	None	RF full model 0.908 (not available)
Niedziela *et al.* (2021) [104]	175 895	6-months	42	ANN, LR	None	None	None	ANN 0.8422 (not available)
Shakhgeldyan *et al.* (2024) [105]	4 677	In-hospital	Not available	LR, RF, SGD	None	None	None	First scenario – 0.85 (not available), second scenario -0.9 (not available)

**Abbreviations:** RF- random forest, SVM - support vector machines, LR - logistic regression, varImp-SBE -variable importance with sequential backward elimination, RFE - Recursive feature elimination, ANN - artificial neural network, DT- decision tree, NB- Naive Bayes, GBC- gradient boosting, XGB -XGBoost, Meta- Meta Classifier, kNN - k-nearest neighbors, GBM- gradient boosting, GNB- Gaussian Naive Bayes, SGD - Stochastic Gradient Descent, LDA - linear discriminant analysis, GRACE - The Global Registry of Acute Coronary Events, TIMI - Thrombolysis in Myocardial Infarction Score, ACTION- Acute Coronary Treatment and Intervention Outcomes Network.

## LIST OF ABBREVIATIONS

ANN: Artificial Neural Network
AUC: Area Under the Curve
BMI: Body Mass Index
BNP: B-type Natriuretic Peptide
CAD: Coronary Artery Disease
CK-MB: Creatine Kinase-MB
CNNs: Convolutional Neural Networks
CRP: C-reactive Protein
DL: Deep Learning
GBM: Gradient Boosting
GNB: Gaussian Naive Bayes
LDL: Low-Density Lipoprotein-C
MI: Myocardial Infarction
ML: Machine Learning
NLR: Neutrophil-to-Lymphocyte Ratio
NT-proBNP: N-terminal pro-brain Natriuretic Peptide
PCA: Principal Component Analysis
PCI: Percutaneous Coronary Intervention
RF: Random Forest
RFE: Recursive Feature Elimination
RNNs: Recurrent Neural Networks
SBP: Systolic Blood Pressure
SMOTE: Synthetic Minority Oversampling Technique
SVM: Support Vector Machines
TC: Total Cholesterol
TG: Triglycerides
UFH: Unfractionated Heparin
